# Monkeypox: a review of epidemiological modelling studies and how modelling has led to mechanistic insight

**DOI:** 10.1017/S0950268823000791

**Published:** 2023-05-23

**Authors:** Marina Banuet-Martinez, Yang Yang, Behnaz Jafari, Avneet Kaur, Zahid A. Butt, Helen H. Chen, Svetlana Yanushkevich, Iain R. Moyles, Jane M. Heffernan, Chapin S. Korosec

**Affiliations:** 1 Climate Change and Global Health Research Group, School of Public Health, University of Alberta, Edmonton, AB, Canada; 2 School of Public Health Sciences, University of Waterloo, Waterloo, ON, Canada; 3 Mathematics and Statistics Department, Faculty of Science, University of Calgary, Calgary, AB, Canada; 4 Department of Biomedical Engineering, Schulich School of Engineering, University of Calgary, Calgary, AB, Canada; 5 Irving K. Barber School of Arts and Sciences, Department of Computer Science, Mathematics, Physics and Statistics, University of British Columbia Okanagan, Kelowna, BC, Canada; 6 Modelling Infection and Immunity Lab, Mathematics and Statistics, York University, Toronto, ON, Canada; 7 Centre for Disease Modelling, Mathematics and Statistics, York University, Toronto, ON, Canada

**Keywords:** climatic variables, mathematical modelling, monkeypox, MPXV, One Health, reproduction number, mpox, mpox vaccination

## Abstract

Human monkeypox (mpox) virus is a viral zoonosis that belongs to the Orthopoxvirus genus of the Poxviridae family, which presents with similar symptoms as those seen in human smallpox patients. Mpox is an increasing concern globally, with over 80,000 cases in non-endemic countries as of December 2022. In this review, we provide a brief history and ecology of mpox, its basic virology, and the key differences in mpox viral fitness traits before and after 2022. We summarize and critique current knowledge from epidemiological mathematical models, within-host models, and between-host transmission models using the One Health approach, where we distinguish between models that focus on immunity from vaccination, geography, climatic variables, as well as animal models. We report various epidemiological parameters, such as the reproduction number, *R*_0_, in a condensed format to facilitate comparison between studies. We focus on how mathematical modelling studies have led to novel mechanistic insight into mpox transmission and pathogenesis. As mpox is predicted to lead to further infection peaks in many historically non-endemic countries, mathematical modelling studies of mpox can provide rapid actionable insights into viral dynamics to guide public health measures and mitigation strategies.

## Introduction

Orthopoxviruses are a genus of viruses that include variola, vaccinia, cowpox, and monkeypox (mpox) viruses. Smallpox, a highly pathogenic orthopoxvirus, is estimated to have claimed the lives of over 300 million people worldwide but was successfully eradicated in 1977 through an international vaccine campaign led by the World Health Organization (WHO). Mpox is endemic to multiple African countries, including Benin, Cameroon, the Central African Republic, the Democratic Republic of the Congo, Gabon, Ivory Coast, Liberia, Nigeria, the Republic of the Congo, Sierra Leone, and South Sudan [1].

**Figure 1.**
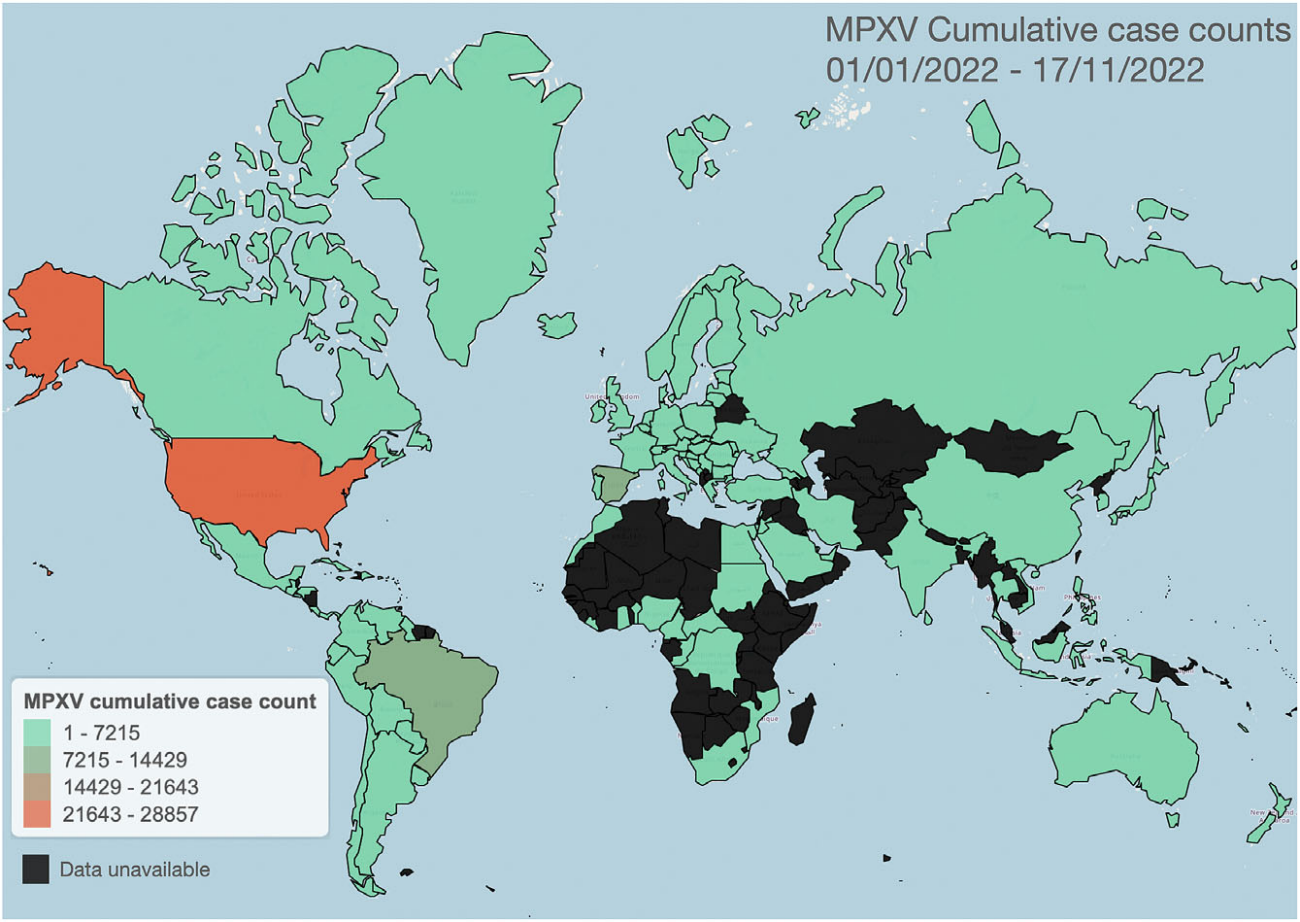
Cumulative mpox cases for the 2022 epidemic from 1 January 2022, through 17 November 2022. Heatmap constructed from publicly available WHO data (ref. [3], accessed 17 November 2022).

**Figure 2.**
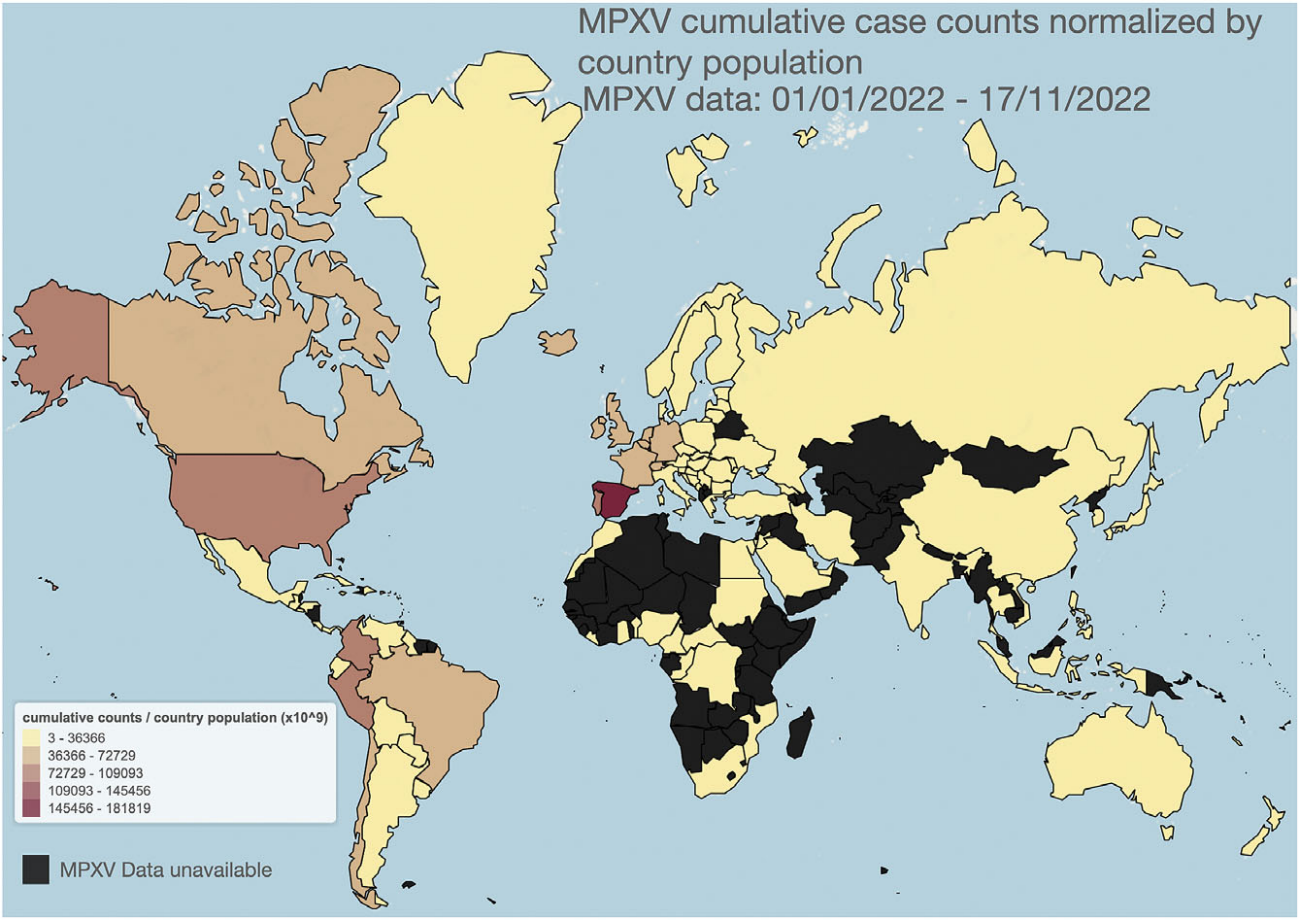
Cumulative mpox cases for the 2022 epidemic from 1 January 2022 through 17 November 2022, normalized by country total population. Heatmap constructed from publicly available WHO data (ref. [3], accessed 17 November 2022). Country population data accessed from WolframAlpha Knowledgebase on 29 November 2022.

Historically, the transmission of mpox in non-endemic regions has been short-lived and contained within a specific geographic area [2]. However, the increased prevalence of mpox in humans since the 1980s has been linked to a decrease in vaccine immunity and an increase in viral fitness traits, making it a significant emerging human threat [2]. In 2022, the World Health Organization (WHO) reported multiple international mpox outbreaks in 20 non-endemic European countries, as well as the United States of America, Canada, Mexico, and much of South America [3]. From May to June 2022, these outbreaks resulted in a total of 780 cases [4]. As of 28 July 2022, the Centers for Disease Control and Prevention (CDC) reported 4907 confirmed cases in the United States, with the total cumulative cases in non-endemic countries exceeding 20,800. By December of 2022, the total reported cumulative cases in non-endemic countries surpassed 80,000 [4]. Figure 1 is a heatmap of global cumulative case counts for the 2022 epidemic as of 17 November 2022. We also include a heatmap of case counts normalized by total country population, shown in Figure 2. In June of 2022, the emergence of mpox in non-endemic countries led the WHO to declare the overall risk of further transmission as ‘moderate’ globally and ‘high’ in the European region. It was hypothesized that mpox mutated to find a new niche in tightly connected sexual networks [5]. As such, mpox now presents a significant public health threat to non-endemic regions, with some countries, such as the United Kingdom, responding by purchasing large amounts of smallpox vaccines for public distribution.

Mathematical modelling has proven to be a valuable tool for understanding epidemics and developing intervention strategies [6, 7]. Modelling of in-host pathogen dynamics has been critical in advancing our understanding of many pathogens, including HIV, HCV, HBV, HSV, influenza, pneumococcus, and SARS-CoV-2, and has also aided the development of vaccine therapies [8–16]. This review provides an in-depth examination of the current epidemiological understanding of mpox from a modelling perspective, and investigates how modelling studies contribute to mechanistic insight into viral fitness and transmission traits. In Section 2, we briefly cover the history and origins of mpox, and in Section 3, we provide an overview of the current basic knowledge of biology and clinical presentation of human mpox. In Section 4, we critique and review population-level modelling studies, differentiating between studies focused on endemic and non-endemic regions, those considering prior immunity from smallpox vaccines, and animal models. We summarize both pre- and post-2022 modelling parameters, including the reproduction number, force of infection, incubation, and recovery rates, in Table 1.

**Table 1.** Table of values listing epidemiological parameters for mpox viral dynamics from the literature

Parameter	Definition	Units	Values (range) [ref.]
Epidemiological mpox parameters in humans			
*R*_0_	Basic reproduction number	N/A	2.13(1.46–2.67) [56], 2.53 (average value)^a^ [69], 2.66 (international estimate)^a^ [50], (1.5–4.3) (Canadian estimate)^a^ [50], 1.5 (high-risk pop.)^a^ [61], 0.01(low-risk pop.)^a^ [61], 2.42–2.88(Spain)^a^ [96], 2.32(UK)^a^ [96], 1.3(international)^a^ [59]
*Rvac*	Disease-free and vaccinated population reproduction number	N/A	0.32(0.22–0.4) [97]
*β*	Infection rate	Days^−1^	1.68x10^−4^ (Canadian estimate) [50]^a^, 9.78x10^−7^(International estimate) [50]^a^
*P_s_*	Transmission probability per sexual contact	N/A	0.24 [61]^a^
*I*	Incubation period	Days	5–21 [55, 56], 8.5(6.6–10.9) [67]^a^, 10–14 [98]
*P*	Prodromal Period	Days	1–4 [31], 2 [98]
*σ*	Timespan from the appearance of lesions to desquamation	Days	14–28 [31], 22–24 [99]
*d_h_*	Human death rate	Days^−1^	3.12 [55]
*Dfrac*	Human infection mortality percentage	%	1–10 [1], 10–17, (from 1970–1989) [98], 1.5 (1997) [98], *<*0.0005 [100]^a^
*βhh*	human-human transmission rate	Days^−1^	32.85 [55]
*ρ_h_*	Human recovery rate	Days^−1^	28.08 [55]
*V_r_*	Optimal vaccination rate	vaccine/yr	0.04 [55]
*Veff*	Cross-vaccine efficacy from smallpox vaccine	%	(80–95) [101]
*Vloss*	Vaccine efficacy loss	%/yr	1.29 [42]
Γ_2_	Secondary attack rate: ratio of infected household members to total household members	%	15 (unvaccinated) [22], 0.4 (vaccinated) [22]
Γ_1_	Primary attack rate: proportion of exposed susceptible population that become ill	%	7.2(unvaccinated) [26], 0.9(vaccinated) [26]
Animal transmission mpox infection parameters			
*d_s_*	Squirrel mpox-related death rate	Days^−1^	17.5 [102]
*ρ_s_*	Squirrel recovery rate	Days^−1^	12 [102]
*βss*	Squirrel–squirrel transmission rate	Days^−1^	40 [55]
*βsh*	Squirrel–human transmission rate	Days^−1^	0.05 [55]

a These values are 2022 epidemic specific; all other values are determined from pre-2022 mpox outbreaks.

## History and ecology of Mpox

A pox-like disease was first reported in 1959 in cynomolgus monkeys and was thus named ‘monkeypox’ [17]. The disease was found to have similar structural features as orthopoxviruses: rectangular with diameter 200–250 *μ*m [17]. It was also observed to present similarly to variola-vaccinia viruses and exhibited a similar serological relationship [17]. Further studies revealed that mpox led to the formation of intracytoplasmic eosinophilic inclusions (small whitish lesions) and could pass serially in rabbit skin [18]. Throughout the 1960s and 1970s, WHO continued to monitor both mpox and smallpox in non-human primates to determine if an animal reservoir existed. In the 1960s, four mpox outbreaks were recorded in animals with no recorded infections in humans [18–20]. In 1966, an mpox outbreak occurred in a zoo and was believed to have been caused by two imported anteaters. The 1966 zoo outbreak had a particularly high mortality rate. Despite containment procedures, mpox spread to nearby enclosures, resulting in 23 animal infections and a total of 11 deaths, including 6 out of 10 infected orangutans [20].

### Transmission between humans

The first human mpox case was reported in 1970 in a 9-month-old baby in the Democratic Republic of Congo [21]. A study of 155 mpox cases in west and central Africa from 1970–1983 estimated only 20% of cases to spread from human-to-human contact, where human mpox cases were primarily suspected to occur from contact with monkeys and squirrels [22]. The human-to-human transmission was noted to ‘stop spontaneously’, with attack rates suspected to be 15% amongst smallpox-unvaccinated households and 0.4% amongst vaccinated [22]. These were noted to be comparably less than smallpox attack rates amongst the unvaccinated which ranged from 33% to 88% [23–25]. A study conducted in Zaire between 1980 and 1984 of 214 patients with human mpox found attack rates for household contacts of 7.2% amongst unvaccinated and 0.9% amongst vaccinated [26]. In this study, 13% of cases were found amongst vaccinated individuals leading to the hypothesis that the immunity gained from smallpox vaccination was waning [26], and further raised a concern that the virus may later become endemic [22].

The low attack rates of mpox, and the unchanging secondary attack rates throughout the 1970s through early 1980s amongst the unvaccinated, supported the decision from the Global Commission to cease the smallpox vaccination programme in Central African countries where mpox was now considered endemic [27]. Multiple self-contained mpox outbreaks were documented through the early 2000s. Of note, a major outbreak in Nigeria began in September of 2017 and ultimately led to 228 suspected cases [28]. Human mpox infections in the 2017 Nigeria outbreak were predominantly male, and the outbreak was ultimately contained [28]. The 2003 mpox outbreak in the USA appeared to be particularly severe in children, where a fifth of paediatric patients developed serious complications resulting in intensive medical intervention, with half of paediatric patients admitted to the ICU [29]. For a detailed review of all pre-2018 human mpox outbreaks, we refer to ref. [2]. The 2022 international emergence of human–human transmission of mpox in multiple non-endemic countries constitutes a significant shift in viral prevalence.

## Pathogenesis, clinical presentation, and longitudinal within-host dynamics of mpox

The incubation period of human mpox can range from 5 to 21 days [30], with a typical incubation period of 7–17 days, followed by a prodromal period of 1–4 days [31]. Clinical characteristics of mpox are similar to those of smallpox: enlarged lymph nodes and a rash period that lasts 14–28 days. Distinct from smallpox, mpox often presents with cervical or inguinal lymphadenopathy, suggesting that the immune response to mpox differs from that of smallpox [31]. A detailed list of clinical characteristics, including changes in mpox epidemiology as a function of time, is described in the article by Wilson et al. [31].

A study on non-human primates longitudinally tracked viral shedding and cytokines from both intrabronchial exposure (i.b.) and intravenous inoculation (i.v.) of mpox [32]. Through tracking mpox viral features over a 36-day window, they found that the time to mean day of lesion exposure increases as a function of decreasing mpox dosage. They further found peak viral load to vary significantly between nasal and oral swabs. Recent clinical human studies in France and Spain have longitudinally tracked cohorts of people over 14 and 57 days [33, 34]. These studies compare mpox viral load between HIV+ and HIV- individuals and find mpox cycle threshold (Ct) values to decrease significantly for both categories of individuals [33, 34], and further conclude transmission of mpox to primarily occur through direct body contact rather than through a respiratory route or bodily fluids [33].

Serological features that inform us about immune responses can also be used by within-host modelling studies to reveal mechanistic insight into viral traits as well as vaccine dynamics. Interferon-gamma (IFNg) is a cytokine known to play a pivotal role in host defence against pathogens [35, 36], and is often used to model within-host inflammatory responses and infer cellular-mediated immunity [15, 16]. Immunity from smallpox vaccination has been shown to elicit IFNg, cytotoxic T cell, and neutralizing protein responses in humans that can last over 20 years [37]. The inflammatory cytokine IFNg has been shown to play an important role in protection against mpox in mice, whereby inactivation of the IFNg receptor led to increased sensitivity to mpox [38]. Earl et al. [38] also report viral titres as a function of time in various major organs, where lungs were found to contain the highest PFU/g for all time points. They also track six cytokines, including IFNg and IL6, as a function of time after injection and find a strong IFNg response in BALB/c mice but not in other types of mice [38]. Interestingly, orthopoxvirus have been shown to suppress recognition of viruses by innate cells through suppressing IFN production [39]. Further, mpox has been found to suppress T-cell activation by triggering a state of T-cell non-responsiveness [40]; thus, a within-host model of mpox should take into account CD4 and CD8 suppression dynamics. These longitudinal data serve as a useful starting point for a within-host modelling study of mpox and can be utilized to guide model predictive power and determine practical identifiability in estimated parameters. Lum et al. [30] provide an in-depth review of the clinical immune features of mpox.

Mpox cross-protective immunity from the smallpox vaccine is known to occur [30]. For example, prairie dogs vaccinated with the smallpox vaccine and then challenged with mpox were found to mount a significant humoral response. Further, vaccinated humans were found to mount strong cellular and humoral responses as shown in longitudinal data over a 32-day study period [41]. However, longer-term studies find efficacy wanes at an approximate rate of 1.29%/yr [42]. We refer to Lum et al. [30] for an in-depth review of mpox clinical immune features.

In the next sections, we review epidemiological modelling efforts of population spread of mpox, distinguishing between human–human, animal–animal, animal–human, and human–animal scenarios. We further cover modelling studies incorporating climate variables, therapeutic strategies (from smallpox vaccine waning and future vaccination outcomes), contact tracing, and isolation measures. Machine learning is emerging as a technology with demonstrated capability for early detection of mpox [43]. However, we do not provide a detailed review of the application of machine learning to mpox; such work can be found in refs. [44, 45].

## Population-level epidemiological models

### SIR/SEIR with no immunity

Compartmental modelling techniques have been used extensively to describe the population spread of infectious diseases. Among infectious disease models, the most fundamental and classic model is the Susceptible-Infected-Recovered (SIR) compartmental model developed by Kermack and McKendrick [46]. In the SIR model, the total population is divided into three subgroups based on the disease status: susceptible (S), infected (I), and recovered (R). S represents the susceptible population that has not yet but may be infected by the disease, I represents the infected population that can transmit the disease, and R represents the population recovered from the infected disease. Two parameters are used in the classic SIR model: the effective contact rate (*β*) and the recovery rate (*γ*). *β* affects the transition from *S* → *I*, and *γ* affects the transition from *I* → *R*, and the total population, *N*, is conserved through time *N*(*t*) = *S*(*t*) + *I*(*t*) + *R*(*t*). An example schematic of the SIR model is shown in Figure 4a.

The epidemiological model framework for mpox has been established over the past few decades, and many models capturing human–human, and animal–human interactions have been explored [47]. Jezek et al. [48] constructed a stochastic model using the Monte Carlo method to simulate the chain of human-to-human transmission of mpox. The model has been validated and applied to understand the transmission potential of mpox in unvaccinated populations [48]. Bhunu and Mushayabasa [49] presented a basic SIR compartmental model to examine the transmission dynamics of mpox between humans and non-humans, and Betti et al. [50] present a SIR model with additional pair-formation dynamics to account for transmission via prolonged close contact between individuals.

We summarize parameters determined by mpox epidemiological modelling studies in Table 1. For the non-human population, mpox parameters are found to be: 2 yr^−1^ for the rate of recruitment for susceptibles, a natural death rate of 1.5 yr^−1^, the death rate due to mpox is given as 0.4 yr^−1^, and the rate of immunity is given as 0.6 yr^−1^ [51]. Pre-2022, for the human population, mpox parameters were found to be: 0.029 yr^−1^ for recruitment rate of susceptibles, a natural death rate of 0.02 yr^−1^, the death rate due to mpox of 0.1–0.17 yr^−1^, and permanent immunity rate of 0.83–0.9 yr^−1^ [51]. The animal-only endemic equilibrium is globally asymptotically stable when *R*_0*n*_ *>* 1 and *R*_0*h*_ *<* 1. The endemic equilibrium, where mpox infections exist in both the human and non-human populations, was shown to be locally asymptotically stable when *R*_0*h*_ *>* 1, but close to 1 [51].

### Models with vaccination

The SIR model often oversimplifies complex disease transmission dynamics. For example, the SIR model does not consider the incubation duration, defined as the span of time between when an individual is exposed to a disease and when that individual becomes infected. We refer to Tolles and Luong [52] who highlight limitations of the traditional SIR model, including that it results in often over-simplified assumptions about the population dynamics. Thus, most epidemiological work involves SIR-inspired models with more mathematical complexity to account for complex population dynamics. The Susceptible-Exposed-Infected-Recovered (SEIR) model has been widely used to study infectious disease dynamics. In the SEIR model, an exposed compartment (E) is added to the fundamental SIR model, representing individuals who are exposed but have not yet been contagious, such that they experience an incubation period. Mitigation strategies such as vaccination can also be considered. For example, Usman et al. [53] developed an SVEIR model (including a vaccinated component) that accounts for a varied incubation period and individual vaccination status. They found that adequate vaccination and treatment policies could dramatically reduce the spread of mpox among humans. Based on mpox parameters prior to year 2017, they conclude that an increase in vaccination control parameters leads to a decrease in the basic reproduction number. Emeka et al. [54] also incorporate a vaccine compartment in a population of mpox-susceptible individuals and generally find that mpox outbreaks do not occur in populations of vaccinated individuals.

**Figure 3.**
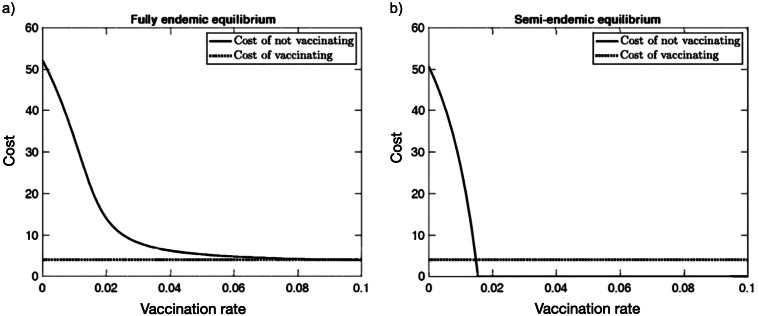
Costs versus vaccination rate with a high rate of the effective human-to-human transmission (*β_hh_* = 60). (a) Fully endemic equilibrium and (b) Semi-endemic equilibrium. Reprinted by permission from PeerJ from ref. [55]. Copyright 2020.

Building on the work of Usman and Adamu [53], Bankuru et al. [55] introduced a simplified SIR model of the mpox dynamics, providing closed-form formulas for equilibrium states of this disease dynamics, allowing for direct calculations of the semi-endemic equilibrium (Figure 3). They showed there exists a semi-endemic equilibrium in which there is no infection in the squirrel population, while the disease still persists in the human population. They found that the optimal vaccination rate amongst humans is about 0.04 vaccine/year, meaning that individuals should be advised to vaccinate approximately once every 25 years. They also found the optimal vaccination rate is about 10 times more sensitive to parameters related to animal hosts than to a corresponding parameter related to humans, thus concluding that more precise information about reservoir hosts is needed [55].

As countries such as the UK are purchasing large quantities of vaccines for public dissemination, given that vaccine efficacy has been found to drop at a rate of 1.29% per year [42], mathematical modelling studies such as that done by Bankuru et al. [55] can be used to inform vaccination rates, as well as which proportion of the population needs to be vaccinated to achieve herd immunity. Another important factor explored by Bankuru et al. [55] is the cost of vaccination. Where cost is defined in a game-theoretic sense, the cost of not vaccinating is given by the product of the cost of infection with the probability of becoming infected. In Figure 3, we include plots of cost as a function of vaccination rate when the human–human transmission rate is high, where Bankuru et al. [55] find that the overall cost of vaccinating is much lower compared to not-vaccinating for most epidemic scenarios.

A combination of historical data and epidemiological modelling was used to estimate the basic reproduction number, *R*_0_, of mpox in the Democratic Republic of the Congo during 1966–1984 to be between 1.46 and 2.17 [56]. Mpox *R*_0_ was thus significantly less than smallpox which had an estimated range of *R*_0_ of 3.2–6.9 [57, 58]. Due to the lasting immunity from the smallpox vaccine, mpox was deemed not self-sustainable in human populations in the DRC from 1980–1984 [56]. However, by the year 2011 estimates show that the immunity from smallpox vaccination against mpox had fallen to 60% in non-endemic countries [56]. Hence, mpox has long been hypothesized to have increasing potential to emerge as an epidemic in humans in historically non-endemic countries.

### Epidemiological modelling studies on the 2022 global outbreak

**Figure 4.**
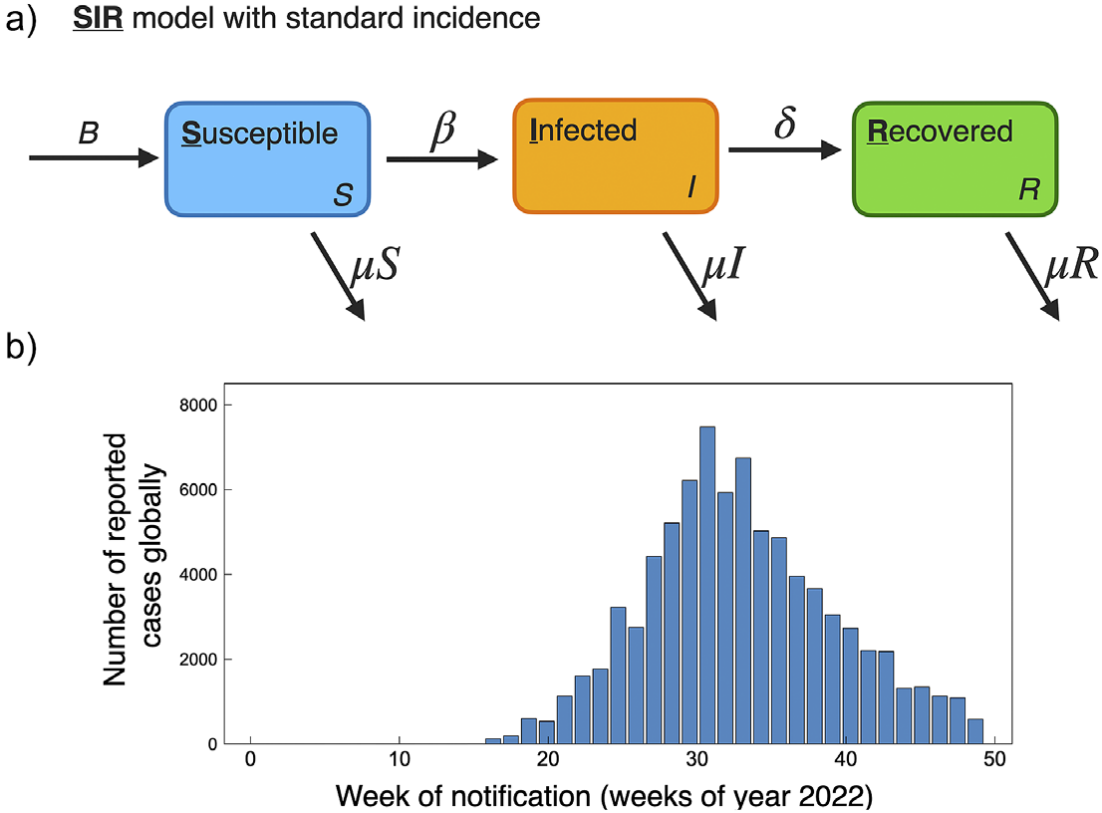
(a) Schematic of basic SIR model with standard incidence, similar as used to model mpox dynamics for the 2022 pandemic [50, 59]. (b) Global reported mpox cases as a function of weeks for the year 2022. Data accessed from publicly available WHO data (ref. [3], accessed 17 November2022).

Population-level human-to-human models of mpox throughout the 2022 epidemic have been largely based on SIR and SEIR frameworks. These modelling studies consider public health mitigation strategies (e.g., quarantine and vaccines), contact tracing, and sexual mixing models. For reference, the current scenario of global cumulative mpox cases by country is shown in Figure 1, and with cases normalized by country population shown in Figure 2. We further include the current global trend as a function of time for 2022, as shown in Figure 4b. We next go through current modelling literature on the 2022 epidemic, and how modelling is bringing further mechanistic insight into mpox dynamics.

The increase in cases from the 2022 mpox global outbreak has been shown to be strongly associated with close intimate sexual contact [33]. For the 2022 epidemic, mpox spread has been predominantly transmitted between men who have sex with men, with one study of 528 diagnosed infections finding 98% of infected persons to be gay or bisexual males [60]. Data-driven individual-level and population-level modelling studies can be used to outline the importance of public health policies and various mitigation strategies. For example, the model presented by Betti et al. [50] presents a novel framework that includes pair formation (accounting for prolonged close contact between individuals) to describe mpox transmission. They show their pair-formation model captures population trends in data with an estimated *R*_0_ of 2.3, and they further predict the occurrence of future waves of infection. Similarly, Bragazzi et al. [61] develop a SEIQR model that includes the sexual behaviour of high-risk individuals and find that *R*_0_ amongst the high-risk population to be ∼1.5, whereas amongst the low-risk population to be as low as 0.007 [61]. Modelling has also been used to disentangle the many factors leading the decline of mpox in the 2022 outbreak. For example, through examining changes in sexual behavioural activity versus vaccination campaigns, studies have found the initial downturn in cases during the 2022 epidemic was largely due to changes in sexual behaviour [62, 63].

Network models have also demonstrated utility towards understanding mpox transmission dynamics. Bisanzio et al. [64] utilize a recently developed individual-based modelling framework [65] whereby they simulate the spread of mpox in a network of 50 million susceptible individuals distributed across *N* cells to represent a population density characteristic of a typical European country of land mass similar to France or Spain. With spread amongst the population driven by an SEIR model, they predict mpox outbreaks lasting 23–37 weeks where mitigation strategies such as contact tracing with isolation followed by vaccination could reduce the median duration of a mpox outbreak by as much as 75%. Another network model by Van Dijck et al. [66] explores the ramifications of undiagnosed mpox cases and predict that if 10% of mpox contacts abstain from sexual activity, this could result in a 35% reduction in cases. Another contact tracing study on the transmission dynamics in the UK predicted the epidemic peak to occur in early July of 2022, and further found that a significant number of cases were caused by pre-symptomatic transmission and determined a mean incubation period of 8.5 days [67].

Compartmental and game-theoretic modelling, as well as modelling infection curves with comparably simple logistic functions, has also proven to be beneficial towards revealing mpox population dynamics and the consequences of various mitigation strategies. Mingione et al. [68] apply the generalized logistic curve to country-wide data from the top 10 non-endemic countries experiencing mpox outbreaks and find agreement with the literature that containment of the outbreak is feasible over the short term if mitigation strategies are employed. Building on previous work similar to ref. [55], Augsburger et al. [69] employ an SVEIR-based model to the 2022 global pandemic and further explore vaccination in a game-theoretic framework where individuals consider cost and benefits to vaccination. They find without vaccination mpox prevalence is predicted to be approximately 3.5 cases per 10^4^ individuals, while with optimal voluntary vaccination, prevalence is predicted to be approximately 0.5 cases per 10^4^ individuals. Thus, vaccination is predicted to be a strong mitigative factor in reducing mpox prevalence and minimizing the chances of mpox becoming endemic is historically non-endemic countries. Savinkina et al. [70] employ an SEIR-based model, utilizing previously published assumptions on low-risk and high-risk population-level reproduction numbers, and simulate the spread of mpox on college campuses. In their hypothetical analysis, they find the absence of mitigation leads to an 83% chance of sustained transmission.

The population modelling studies of the 2022 global outbreak all agree, based on current data on mpox trends, that the outbreaks occurring in non-endemic countries are generally under control and on a declining trend. This is of course supported by the current global trend in cases; a histogram of global case counts up to 29 November 2022 is shown in Figure 4b. A summary of 2022 mpox mathematical modelling population parameters is provided in Table 1.

Epidemiological modelling studies are important for policy decision-makers when deciding which mitigation strategy or control measures (such as isolation and lockdown measures) to employ. Predictive modelling for future mpox peaks will be important in aiding policy decision-makers. For example, modelling techniques on mpox have been developed to estimate the true number of unreported cases, and further, have shown promise to accurately predict infection cycles [71].

Modelling studies clearly highlight the important of mitigation strategies. For example, vaccination campaigns should be organized to reduce population infectivity and further reduce the probability of allowing a more virulent and transmissive mpox strain to emerge. Yuan et al. [72] consider an SEIR model whereby the population is divided into high and low risk and focus their study on mass gathering scenarios. They find that a broad vaccination campaign is less effective in curbing the spread of mpox than compared to contact tracing, isolation, and vaccination of close contacts. They further posit that the ring vaccination strategy may be inadequate in preventing an outbreak from occurring; however, it does still result in fewer case counts [72]. They follow up their work with a study to consider the mpox threat to the low-risk population if viral transmissibility increases [73]. They conclude that isolation, contact tracing, and quarantine are key mitigation strategies to prevent infection in the event of increased viral transmission into low-risk populations [73]. Predictive modelling for future mpox peaks can be an important factor in aiding policy decision-makers. For example, based on Canadian mpox trends, there are predicted to be further peaks occurring on an approximately annual basis [50].

As noted, the 2022-mpox strain is predominantly spreading through close intimate contacts [33]. However, orthopoxviruses, such as smallpox, are known to transmit via a respiratory route [74]. Currently, a respiratory transmission mode is not found to play a major role in the 2022 outbreak [33, 34]. Thus, modelling studies, such as bottleneck studies [75], that aim to predict mutation lineages and probabilities of mutant transmission, can play an important role in predicting the potential severity of future mpox mutants. The concern that mpox could mutate to find a respiratory transmission route is warranted. The cost and benefit of mitigation strategies, including the details of how they can be disseminated to the public, can be readily explored through modelling studies to aid public health campaigns should the virus emerge with a more infectious mutant.

### Immunity decline hypothesis

**Figure 5.**
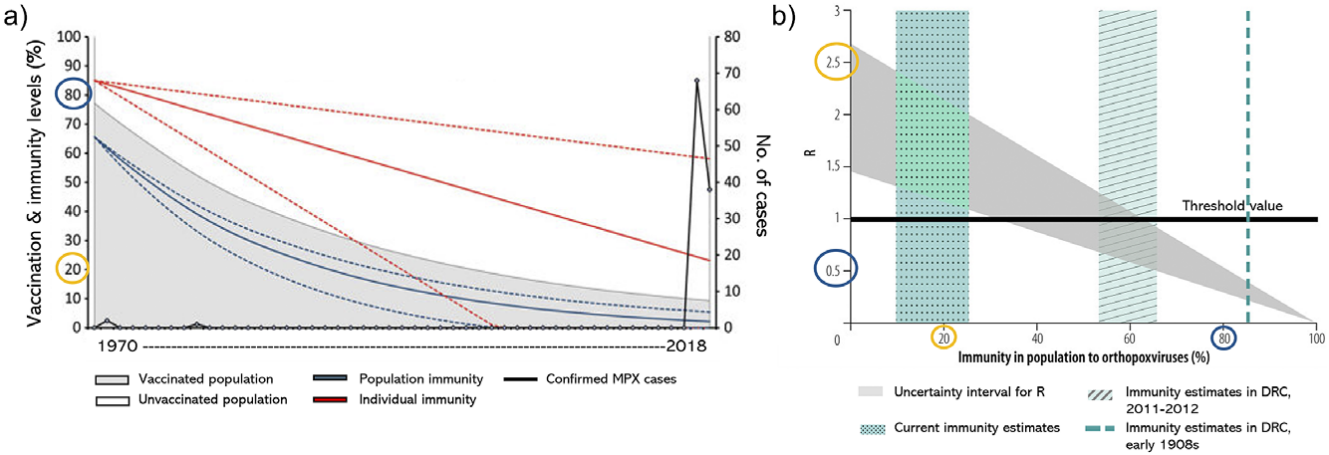
(a) Visualization of the relationship between smallpox vaccination and cross-immunity conferred to mpox virus rates at a population (blue) and individual level (red) in Nigeria from 1970 to 2018. Reprinted by permission from Centers for Disease Control and Prevention from ref. [42]. Copyright 2021. (b) Predicted change of the reproduction number R for MPX as a function of immunity in a population to orthopoxvirus species (provided by smallpox vaccine). Blue circles show a scenario where the vaccination percentage is high, most of the population presents high-level cross-immunity against orthopoxvirus species, and the mpox R value is low. Yellow circles show the scenario where vaccination and cross-immunity rates are low, and mpox R-value increases to *>*2.5. Reprinted by permission from the World Health Organization from ref. [56]. Copyright 2020.

The recent 2022 emergence and outbreaks of mpox are still under investigation. One hypothesis for the increase in cases relates to the decline in population cross-immunity provided by the smallpox vaccine [76, 77]. In 1980, the WHO declared the eradication of smallpox. Soon afterwards, routine smallpox immunization ended worldwide [78]. Smallpox vaccine has proven to induce humoral and cell-mediated responses against orthopoxviruses [79, 80], creating a heterotypic immunity composed of a wide array of antigen receptors [81] and estimated to have an efficacy of 85% in preventing mpox infection in humans [56]. Thus, it has been suggested that younger generations not vaccinated against smallpox are vulnerable to mpox infection. This section will discuss the current evidence from mathematical models testing the declining immunity from vaccination in increasing susceptibility to mpox. Data from the Democratic Republic of the Congo (DRC) revealed that individuals born before the official vaccination cessation had a 5.21-fold lower risk of mpox infection than unvaccinated persons [76, 82]. Nguyen et al. [83] modelled the declining immunity in Nigeria, accounting for individual-level declining immunity at a rate of 1.29% per year, as well as country-wide declining immunity using weighted regional estimates of smallpox vaccination coverage. They found the increase in unvaccinated and immunologically naive population (90.7% of the total population in Nigeria in 2018), and together with the decline from 85% to 23.1% in efficacy from cross-immunity protection provided by smallpox vaccination, and that the overall population immunity was estimated to be only 2.2% as of 2018 [42]. Shown in Figure 5a, we include an example of their findings.

The declining immunity from vaccination to smallpox represents an epidemiological threat by increasing the mpox reproduction number. The basic reproduction number, R_0_, of any infectious disease is dynamic and depends on many variables, including characteristics of the pathogen characteristics and the host. Grant et al. [56] modelled this relationship with data from the DRC. They determine an mpox reproduction number, *R.* *R* is given by *R* = *R*_0_(1 − *ϵp*), where *ϵ* represents the vaccine efficacy, and *p* the vaccination coverage. Given a current immunity estimate, they determine *R* could be higher than 2.5 [56]. We include a plot of their results for *R* as a function immunity in Figure 5b.

The increase in attack rate over time may be evidence for the immunity decline hypothesis as well. Mpox household attack rates amongst the unvaccinated and vaccinated were reported as 15% and 0.4%, respectively, in 1985 [22]. The 2013 outbreak in the DRC, which represented a 600-fold increase in annual infections, was found to have a household attack rate of 50%, where many people who contracted mpox were previous smallpox vaccine recipients [84].

The loss of immunity hypothesis is not mutually exclusive from other re-emergence theories, such as the increased exposure to wildlife, reservoir expansion, globalization, and mutations to mpox fitness traits. These factors represent critical barriers to consider for mpox spillover opportunity [85]. An increase in the mpox immune-naive population and the risk of exposure create a niche for continued mpox animal-to-human and human-to-human transmission, longer chains of infection, and thus an opportunity for mutation in mpox viral transmission traits. Pre-2022, human-to-human transmission chains have been relatively short-lived, and stochastic models performed in the 1980s based on historical data found mpox to have a low probability to be established in human populations [48]. However, more recent models have shown that sustained human-to-human transmissions could favour pathogen evolution, creating a potential existence of semi-endemic or fully endemic equilibrium [56, 86].

A clustered epidemiological differential model developed by Ali et al. [87] considered human behavioural dynamics such as vaccination and drug hesitancy, cooperation, and mobility rate and showed how opination dynamics have a tremendous impact on fatality rates. Furthermore, models on voluntary vaccination have shown the potential control of mpox outbreaks in a semi-endemic equilibrium but not in a fully endemic one [55]. In an endemic equilibrium scenario, deterministic compartmental models showed that isolation of infected individuals, in combination with adequate treatment and vaccination, plays an essential role in the control and eradication of mpox [53, 88]. Modelling efforts show vaccination remains a high-potential primary mpox mitigation strategy and should continue to be prioritized in endemic regions [76]. However, to achieve effective mpox management, a combination of countermeasures needs to be considered. Novel mpox-specific vaccines [89, 90], treatments [91–93], and prophylaxis public health measures [87, 94, 95] are all under development to mitigate mpox spread.

### Effect of reservoirs and wildlife control measures

A report by the WHO in 1968 concluded that mpox transmission between monkeys is ‘infrequent’ and that most likely another animal reservoir existed [18]. A definitive mpox virus reservoir host is still unknown and under study. Currently, giant-pouched rats, rope squirrels, and African dormice are posited as the most likely candidates [55, 103]. Throughout the 1980s, the animal–animal spread was found with particular prevalence in squirrels of the *Funisciurus anerythrus* species, where it was shown they sustain mpox viral transmission in areas near human settlements [104]. Squirrel mpox-related death rates and recovery rates were later found to be approximately 17.5 and 12 days, respectively [102] (see Table 1).

During the 2022 global mpox outbreak, it was discovered that human-to-dog transmission is possible, thus raising concerns about further dog-to-dog and dog-to-human transmission [105]. Culling, the reduction in wild animal populations through selective slaughter, has been employed as a method for wildlife reservoir management and to mitigate the potential of further animal-to-human transmission [106]. For example, culling has been employed recently during the SARS-CoV-2 pandemic to mitigate further animal-to-animal transmission amongst farmed minks [107]. Culling to prevent further mpox spread has been explored through transmission modelling approaches, where it has been found to be ineffective and can lead to the counter-productive outcome of increasing mpox infection. This is because culling results in the sudden removal of mature animals with immunity replaced with juvenile, more susceptible animals, thus increasing the probability of outbreaks [108].

### Climatic variables influencing mpox transmission: a One Health approach

The One Health approach aims to recognize the strong linkage between the health of humans, animals, plants, and the environment, to develop integrated and sustainable solutions [109]. Given the interconnected coexistence between humans, animals, and the environment, mpox emergence in the context of climate change represents a One Health challenge [110, 111]. From a One Health perspective, we present current evidence on mathematical modelling connecting climate change impacts on the environment, animals, and humans, to mpox dynamics.

Climate change has altered human–environment systems [112]. The emergence and re-emergence of many infectious diseases are projected to increase due to the negative impact of climate change [113, 114]. Interactions between the three factors embodied in the epidemiological triangle: the virus (agent), the human (host), and the reservoir (environment) [115], have been found to contribute to mpox emergence and expansion. In addition to the decrease in herd immunity caused by the cessation of smallpox vaccination (discussed in detail in Sections 4.2 and 4.4), climatic variables and human behaviour have created an ideal niche for mpox transmission [116, 117]. In this section, we discuss the current model-based evidence for mpox transmission, emphasizing the influence of climate factors.

The impact on human health from climate change is an emerging topic. There is a consensus on increased adverse climate-related health outcomes such as food insecurity, heath-related mortality and morbidity, mental health damage, or injuries [112]. Impacts on health can include the impairment of the immune system due to direct or indirect effects of climate change.

There has been significant scientific interest in mpox spread within endemic African countries with particular attention to mpox biogeographic barriers [121]. Environmental conditions can define the spread and durability of pathogens outside their hosts. Survival models have shown that orthopoxviruses are high-virulence high-survival pathogens, which implies high durability outside their host [122]. Seasonal patterns of mpox outbreaks have been observed during the fall season and linked to deforestation and flooding [123]. Historical evidence suggests that dense and humid lowland tropical forests ecotones are the most favourable ecosystem for zoonotic transmission of mpox [120, 124].

**Figure 6.**
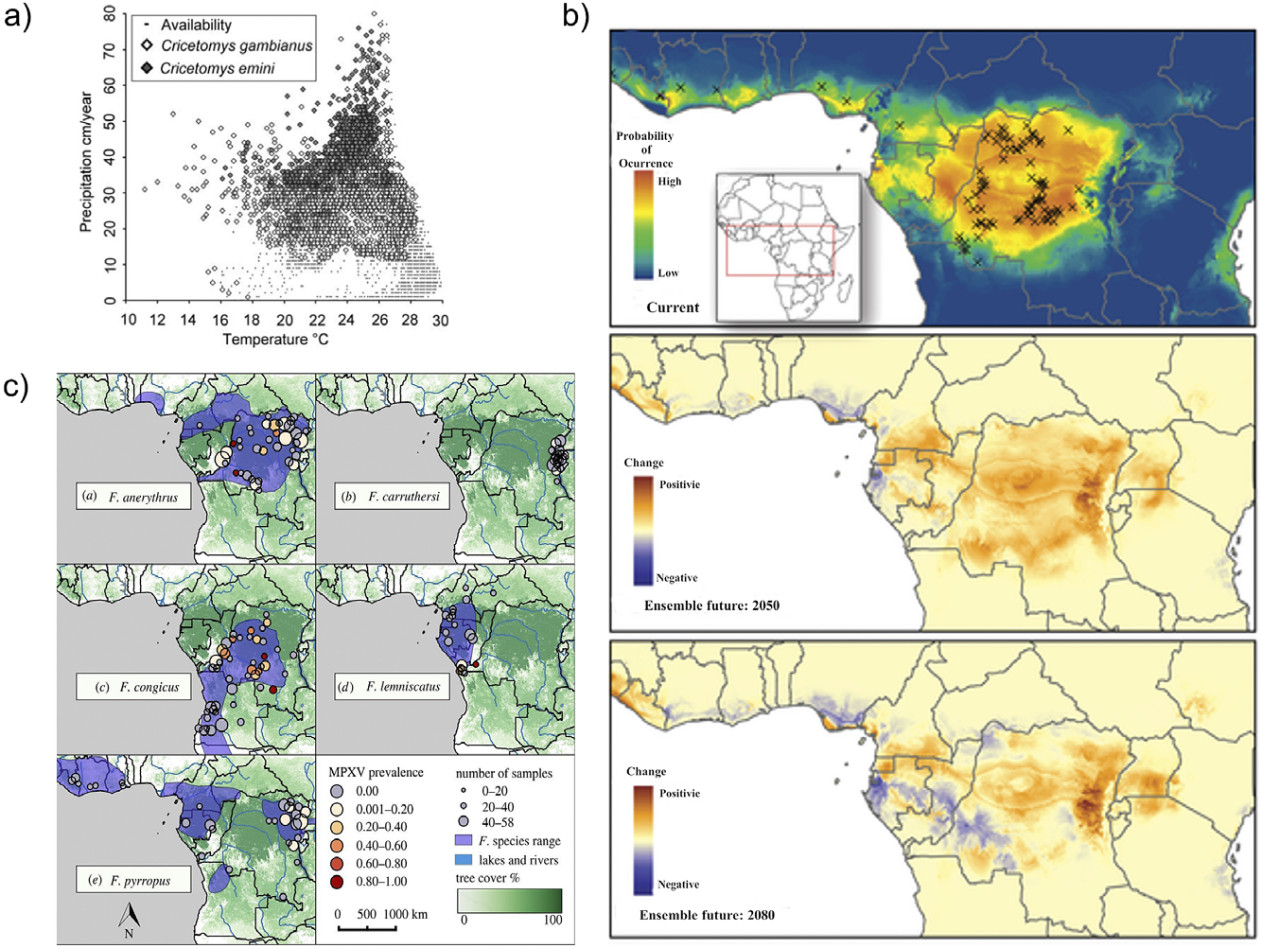
(a) Two-dimensional representation (annual mean temperature and annual mean precipitation) of ecological niche models developed for two mpox reservoir species *cricetomys gambianus* (white diamonds) and *cricetomys emini* (grey diamonds) across tropical sub-Saharan Africa. Reprinted by permission from Oxford University Press from ref. [118]. Copyright 2006. (b) Observed and predicted human mpox occurrence under present and future climate conditions with reservoir species as predictor variables in Central and Western Africa. The average projected change in occurrence probability for eight climate change scenarios for 2050 (middle) and 2080 (bottom). Reprinted and modified by permission from PLOS One from ref. [119]. Copyright 2013. (c) mpox prevalence detected in dried museum specimens of potential mpox reservoir species, with an underlying layer representing tree cover, with darker greens corresponding to high cover percentages. Reprinted by permission from The Royal Society Publishing from ref. [120]. Copyright 2018.

Prior to the 2022 outbreak, mathematical models concluded that continued mpox human–human population spread required continued zoonotic reservoir exposure to maintain chains of transmission [27]. Therefore, much attention has been paid to mpox reservoirs; however, there is no clear consensus on the natural or definitive reservoir as of the time of writing [116, 125]. It is known that environmental conditions can affect the transmission of mpox between animals [126]. Having an unknown primary reservoir for mpox limits a model’s accuracy in the prediction of the impact of climate variables on the animal–animal and animal–human dynamics [127]. Multivariate analyses of historical data have demonstrated that mpox can co-occur on several species in an unanticipated manner [120, 128]. Additionally, ecological niche modelling techniques have been used to model the climate and spatial distribution of mpox [118, 128, 129], where these modelling studies emphasized the critical role of ecosystem variation on reservoir distribution (shown in Figure 6).

Understanding mpox spatial ecology is essential to predict future outbreaks under climate change conditions. Spatial and probabilistic models have been used to study mpox occurrence, particularly in Africa (see, e.g., Figure 6b,c). Including climatic variables has been demonstrated to be critical in the spatial analysis of mpox at a local scale and regional scale [119, 130]. Climatic variables such as temperature and precipitation seasonality are reservoir species predictors, meaning that a small change in those variables could also change the reservoir dynamics and thus animal–human transmission probability [118, 119]. Furthermore, climatic and ecosystem variables can increase habitat suitability for potential mpox reservoirs and, by extension, more frequent wildlife–human contact [124, 128]. Other extreme weather events, such as droughts [131], can force carrying mpox species to move closer to human settlements [119]. Future research predicting shifts in reservoir species should also focus on how this dynamic is affected by environmental changes. We propose that models should include the viral dynamic considerations of interrupting or increasing wildlife–human interaction frequency under climate change scenarios.

### Wastewater-based epidemiology to monitor mpox levels

Wastewater-based epidemiology (WBE) is a population-level biomarker surveillance method to analyse wastewater for either chemicals or pathogens [132, 133]. WBE has been shown to be able to estimate mpox population trends through time; however, key gaps in our understanding of the application of WBE to mpox have been highlighted, namely, the current lack of a 100% inclusive WBE methodology and limits cross-reactivity with non-targeted species [134]. Towards applying WBE to mpox, Chen and Bibby [135] developed a Monte Carlo approach to estimate the probability of detecting mpox DNA in wastewater. They determine that United States wastewater treatment plants may be able to detect 7 infections out of 100,000 people based on previously reported daily shedding rates.

### Towards a within-host model for mpox

The goal of within-host modelling is to represent the complex physiological processes of a disease, or therapeutic, within the body with mathematical models [7]. Within-host mathematical models are developed under biological principles and then fit longitudinal serological data to estimate various aspects of physiological dynamical outcomes. Modelling of in-host pathogen dynamics has proven critical towards furthering our understanding of HIV, HCV, HBV, HSV, influenza, and SARS-CoV-2 as well as aiding the development of vaccine therapies [8–12,14–16,136–139]. Following the development and fitting of a model to serological data, structural and practical identifiability methods are then employed to assess model reproducibility and reliability [140]. Within-host models have been used extensively to estimate properties of disease dynamics, thus contributing to our understanding of the disease progression at the within-host scale [12,75,137,141–146].

At the time of writing, there is a noticeable lack of within-host mechanistic modelling studies of mpox, with few within-host studies for any orthopoxvirus. The work of Ogunjimi et al. [147], who model the CD4 trajectories of human chickenpox, to the best of our knowledge, is the only published orthopoxvirus within-host modelling work. The mpox longitudinal clinical studies outlined in Section 3 provides an overview of current knowledge of mpox serological parameters required to fit to a typical within-host model and should serve as a strong starting point for such a study.

## Future directions and concluding thoughts

Mathematical modelling provides a cost-effective and non-invasive methodology for gaining actionable insights into viral dynamics and therapeutic responses at the population and within-host levels. At the within-host level, mathematical modelling utilizes serology-based diagnostics to understand disease transmission dynamics, including viral reproduction numbers, viral load clearance, and cell recovery, to understand the timescales of disease transmission.

However, such studies on mpox are currently limited. At the population level, mathematical modelling leverages population metrics, such as contact tracing data, cumulative case counts, and wastewater surveillance, to predict outbreak characteristics such as recovery rates, transmission, virulence, and reproduction numbers. Although the current mpox epidemic case counts are declining, models predict future waves to occur annually [50]. Therefore, modelling efforts can assist in the allocation of public health resources to mitigate the future spread of infection, such as identifying when and whom to target in vaccine or education campaigns.

The burden of human infectious disease remains high in many countries, with recent outbreaks of emerging and re-emerging pathogens referred to as the ‘new era of infectious disease’ [148]. Climate change is causing significant changes in natural ecosystems worldwide [112]. More than half of infectious diseases affecting human populations having been aggravated by climate hazards through pathways such as bringing pathogens closer to people or causing favourable changes to viral fitness traits [149]. Mathematical models of infectious diseases that consider climatic variables, such as accomplished for diseases such as influenza virus [150], West Nile virus [151], SARS-CoV2 [152], and Malaria [153], have demonstrated utility for policymakers in planning public health prevention and responses strategies [154]. This review revealed that the practice of including climatic variables in the mathematical modelling of mpox still needs further exploration. For example, current modelling evidence suggests that climate variables can significantly impact mpox transmission and pathogenesis by affecting the reservoir–human contact environment [118–120]. Therefore, it is crucial to consider climatic variables at the local, regional, and global scales in future mpox mathematical modelling studies to better understand its complex dynamics with potential reservoirs and potential impacts on human populations.

The emergence of mpox as a global threat in 2022 has resulted in over 80,000 cases in non-endemic countries as of 17 November 2022. As mpox has gained global attention, it is becoming increasingly important to conduct higher resolution studies that report regular case counts and longitudinal serological measures, such as IgGs, and CD4/CD8 responses, which can be utilized in mathematical modelling approaches to gain deeper insight into viral dynamics and predictive power. An interdisciplinary work between clinicians and mathematicians can better inform timescales of clinical data acquisition to gain the optimal information on disease dynamics from limited data sets [15]. To date, no within-host modelling studies of mpox have been carried out to our knowledge.

Efforts to quantify an immunological correlation of protection in humans against mpox have been reported [41]. However, a robust correlate of protection against the 2022 strain still needs to be discovered [30]. Mathematical approaches can leverage serological studies to correlate humoral and cellular longitudinal responses with case severity or vaccine efficacy, similar to what has been done for SARS-CoV-2 [16]. It is also important to understand differences in within-host dynamics amongst cohorts containing various comorbidities, notably high-risk individuals co-infected with syphilis or HIV [155]. Longitudinal studies working to understand the risks of vaccination in these vulnerable populations need to be included. As has become evident throughout the SARS-CoV-2 pandemic, many long-term consequences of SARS-CoV-2 can present as neurological or psychiatric [156], cardiovascular [157], and various immunological dysfunctions [158]. Longitudinal studies to identify and understand the extent of these potential long-term consequences for moderate and severe mpox cases will become increasingly important. Mathematical modelling can help predict the proportion of individuals expected to suffer from long-term consequences of mpox infection and inform public health policy decisions.

## Data Availability

Sources for all plotted data have been referenced within the figure captions. Data are available from Chapin S. Korosec, Email: chapinSkorosec@gmail.com.
